# The Role of Muscle Spindle Feedback in the Guidance of Hindlimb Movement by the Ipsilateral Forelimb during Locomotion in Mice

**DOI:** 10.1523/ENEURO.0432-21.2021

**Published:** 2021-11-24

**Authors:** William P. Mayer, Turgay Akay

**Affiliations:** Atlantic Mobility Action Project, Brain Repair Centre, Department of Medical Neuroscience, Dalhousie University, Halifax, Nova Scotia B3H 4R2, Canada

**Keywords:** electromyogram, locomotion, mouse genetics, muscle spindles, proprioception, stumbling corrective reaction

## Abstract

Safe and efficient locomotion relies on placing the foot on a reliable surface at the end of each leg swing movement. Visual information has been shown to be important for determining the location of foot placement in humans during walking when precision is required. Yet in quadrupedal animals where the hindlimbs are outside of the visual field, such as in mice, the mechanisms by which precise foot placement is achieved remain unclear. Here we show that the placement of the hindlimb paw is determined by the position of the forelimb paw during normal locomotion and in the presence of perturbations. When a perturbation elicits a stumbling corrective reaction, we found that the forelimb paw shifts posteriorly relative to body at the end of stance, and this spatial shift is echoed in hindlimb paw placement at the end of the swing movement. Using a mutant mouse line in which muscle spindle feedback is selectively removed, we show that this posterior shift of paw placement is dependent on muscle spindle feedback in the hindlimb but not in the forelimb. These findings uncover a neuronal mechanism that is independent of vision to ensure safe locomotion during perturbation. This mechanism adds to our general knowledge of how the nervous system controls targeted limb movements and could inform the development of autonomous walking machines.

## Significance Statement

Safe locomotion relies on placing the foot on a reliable surface at the end of each swing of the leg. During human walking, this foot placement is determined by vision. However, in quadrupedal mammals the hindlimb is outside of the visual field, suggesting that, as in insects, swing movements of the posterior legs might be guided by the anterior legs using proprioception. Here we show that the regularity and precision of hindlimb foot placement in mice are guided by proprioceptive sensory feedback from muscle spindles, contributing to robust locomotion even in the face of perturbations.

## Introduction

Terrestrial locomotion depends on the temporal and phasic coordination of limb muscle activity, driven by stereotypic and individualized patterns of flexor and extensor muscle activation at specific joints (Engberg and Lundberg, 1969; Grillner, 1981; Rossignol, 1996). At the spinal level, core aspects of locomotion are thought to emerge through the actions of interneuron circuitry, referred to as to the “central pattern generator” (CPG), which is capable of generating alternating motor output in the absence of descending or sensory feedback. In addition, sensory feedback mediated by cutaneous and proprioceptive inputs are known to be critical for shaping CPG activity to produce functional locomotion in natural environments (McCrea, 2001; Pearson, 2004; Rossignol et al., 2006; Büschges et al., 2008). Thus, naturalistic locomotor patterns that are robust enough to account for variability in the environment are produced by integrated functions of the CPG and sensory feedback. However, which aspects of locomotor behavior are controlled by sensory feedback remain obscure.

Placing the foot on a secure surface at the end of swing movement is required for robust locomotion and relies on sensory feedback. Previous research in humans has provided evidence that, during walking, visual information is used to determine the precise location of foot placement following a swing movement (Reynolds and Day, 2005). Furthermore, visual information is essential during a critical time window in the latter half of the preceding stance phase, but it is not continuously used throughout the swing (Matthis et al., 2015, 2017). These findings suggest that following the visual determination of where to target the foot, movement is initiated and the foot moves throughout the swing with inertial motion (Matthis et al., 2015). Moreover, corrections of the swing can be made during an ongoing movement with a reaction time of ∼120 ms (Reynolds and Day, 2005). However, in quadrupeds, visual information is less likely to guide the hindlimb as the animals typically look forward and the hindlimb is positioned outside of the visual field. Furthermore, feedback control limited to a “critical time window” would not work in mice because their lighter legs make inertial motion (ballistic movement) less likely (Hooper et al., 2009; Hooper, 2012). In stick insects that have relatively light legs, it has been shown that the anterior legs guide the positioning of posterior legs (Cruse, 1979; Dean and Wendler, 1983; Wosnitza et al., 2013), and this process is controlled by proprioceptive sensory feedback (Brunn and Dean, 1994; Theunissen et al., 2014). We hypothesized that mice also compensate for a lack of visual information by guiding their hindlegs based on the position of the forelimb during locomotion, and this guidance is controlled by displacement-sensitive proprioceptive feedback from muscle spindles.

Previous research measuring accurate foot placement in mice during locomotion on a horizontal ladder suggests that proprioception is required for foot placement in quadrupeds (Akay et al., 2014; Takeoka et al., 2014; Vincent et al., 2016). However, whether a similar mechanism is present for more typical locomotion on a flat surface, and how precision is achieved even in the face of perturbations is not known. We set out to determine whether targeting mechanisms that have been shown in insects also exist in quadrupedal mammals during normal locomotion (Cruse, 1979; Dean and Wendler, 1983; Wosnitza et al., 2013). To address this question, we measured spatial kinematic parameters in wild-type mice and in mutant mice that lack muscle spindles and, therefore, do not receive proprioceptive displacement signals during locomotion without and with perturbations. Focusing on the positioning of the hindlimb paw at the end of the swing movement relative to forelimb paw position at the end of forelimb stance, we found that the movement of the hindlimb is guided by the forelimb. Furthermore, we show that this guidance mechanism is dependent on proprioceptive sensory feedback from muscle spindles. These observations resemble previous findings in insects, pointing to an evolutionarily common mechanism for robust locomotion.

## Materials and Methods

### Animals

Experiments were conducted on four wild-type mice (one male and three females) and six *Egr3-KO* mice (two males and four females; Tourtellotte and Milbrandt, 1998). The data obtained from male and female mice were pooled together as there was no detectable qualitative or quantitative difference between sexes within each group. None of the mice were trained before the experiments. All procedures were conducted in accordance with the Canadian Council on Animal Care and were approved by the University Committee on Laboratory Animals at Dalhousie University.

### Surgeries

The right hindlimbs of adult mice were implanted with bipolar EMG recording electrodes and nerve stimulation electrodes (Mayer and Akay, 2018). Briefly, mice were anesthetized with isoflurane, ophthalmic eye ointment was applied to the eyes, and the skin of the mice was sterilized with a three-part skin scrub using HIBITANE, alcohol, and povidone-iodine. A custom-built set of five EMG recording electrodes and one nerve stimulation cuff electrode were implanted as follows. The neck region and the hindlimbs were shaved. Small incisions were made in the skin at the neck area and at the hindlimbs just above the muscles from which the recordings were made. The electrodes were led under the skin from the neck incision to the leg incisions and implanted into different flexor and extensor muscles that move different leg joints. The EMG electrodes were implanted into the hip flexor [iliopsoas (Ip)], knee flexor [semitendinosus (St)] and extensor [vastus lateralis (VL)], and the ankle flexor [tibialis anterior (TA)] and extensor [gastrocnemius (Gs)] muscles. The nerve stimulation electrode was attached to the saphenous nerve. The skin incisions on the leg were then closed with sutures, and anesthetic was discontinued. Analgesics, buprenorphine (0.03 mg/kg) and ketoprofen (5 mg/kg), were injected subcutaneously 1 h before the surgery. Additional injections were performed at 12 h intervals for 48 h. After surgery, mice were housed separately, placed in a warmed cage with a fresh mass of hydrogel for the first 3 d, and then returned to their regular mouse rack. Any handling of the mouse was avoided until mice were fully recovered, and the first recording session started at least 10 d after electrode implantation surgeries.

### Behavioral recording sessions

Following the full recovery from electrode implantation surgeries, the behavioral recordings were performed as previously described (Mayer and Akay, 2018). Under brief anesthesia with isoflurane, custom-made cone-shaped reflective markers (diameter, 1–2 mm) were attached to the skin at the level of the anterior tip of the iliac crest, hip, knee, ankle, the metatarsal phalangeal joint, and the tip of the fourth digit (toe). Mice were then placed on a mouse treadmill (model MA 102; custom built in the workshop of the Zoological Institute, University of Cologne, Cologne, Germany) following the discontinuation of the anesthesia. The electrodes were connected to an amplifier (model 102, custom built in the workshop of the Zoological Institute, University of Cologne) and to a stimulus isolation unit (ISO-Flex). At least 5 min were given for the recovery from anesthesia. Before any behavioral measurements were made, the saphenous nerve was electrically stimulated with two brief impulses (duration, 0.2 ms; frequency, 500 Hz) with gradually increasing stimulation strength to identify the minimum current that was necessary to elicit reliable muscle response. Following the determination of this threshold current (*T*), the current was set to 1.2 × *T* for the rest of the experiment, which varied between 96 and 1800 μA from animal to animal. The speed of the treadmill was set to 0.3 m/s. When the treadmill was turned on, the mice started walking. We first collected control steps by letting the mice locomote without nerve stimulation. After this initial period, the saphenous nerve was stimulated every 2 s with five pulses (duration, 0.2 ms; frequency, 500 Hz) while mice were moving to introduce a perturbation. When the stimulation occurred while the hindlimb was performing a swing movement, a stumbling corrective reaction (SCR) was elicited. For the “cautions walking” steps, three steps before the SCRs that were not exposed to stimulation were taken as previously described (Santuz et al., 2019). Mice were filmed from the sagittal plane with a high-speed video camera (model IL3, Fastec Imaging) at 250 frames/s, and video files were stored on a computer for later motion analysis. The EMG data were stored separately on the computer by using the Digitizer (model Power 1401, Cambridge Electronic Design) combined with spike2 software (version 8, Cambridge Electronic Design).

### Data analysis

The kinematic parameters of stepping were obtained from the video files using Motus Vicon or custom-made software written by Dr. Nicolas Stifani with ImageJ (KinemaJ) and R (KinemaR; Bui et al., 2016). The coordinates and the angular joint movements were then imported into the spike2 files containing the EMG data and the kinematic and EMG data were synchronized using a custom-written spike2 script. Data analysis was performed using this final spike2 file containing the merged EMG and kinematic data. In the results, all average values are denoted along with the SDs. All plots were made using Excel 2016 software, and statistical analyses were performed with the data analysis package for Excel (statistiXL, version 1.8). Statistical comparisons of averages were performed using Student’s *t* test, and the differences in variabilities were tested with an *F* test, unless otherwise stated.

## Results

### General structure of the swing movement during locomotion and the role of muscle spindle feedback

**Figure 1.**
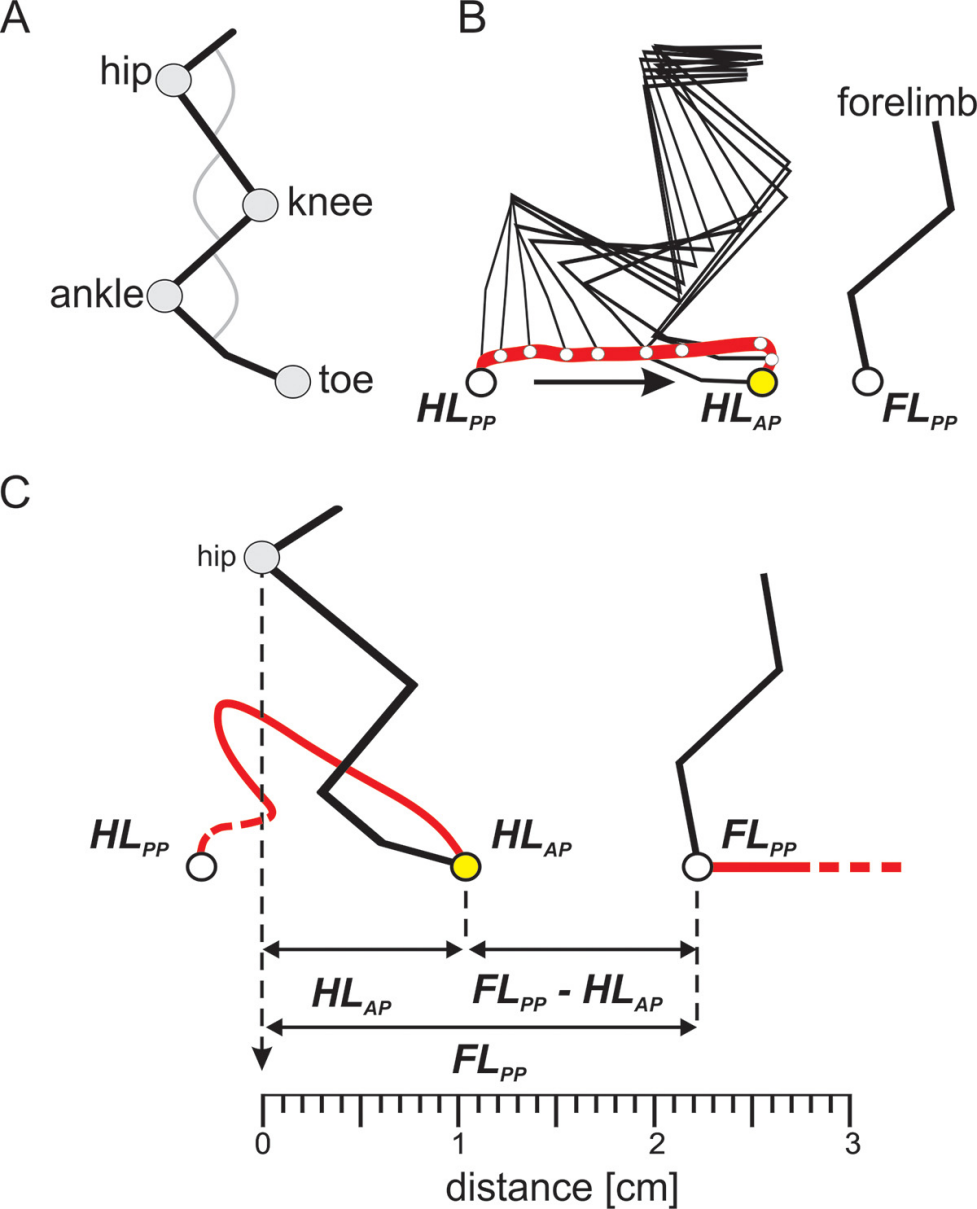
Schematic representation of precise foot placement assessment during locomotion on the treadmill. ***A***, Kinematic data obtained by reconstruction of the hindlimb by means of detecting the coordinates of markers attached to the skin over imposed hindlimb segments. ***B***, Stick diagrams reconstructed by connecting marker coordinates showing the kinematics of a swing phase while the hindlimb is traveling from a posterior position (HL_PP_) to an anterior position (HL_AP_), alongside coordinates of the posterior position of the forelimb (FL_PP_). ***C***, Hindlimb targeting obtained by measurements of distances between toe to the hip joint (HL_AP_), forelimb paw to hip joint (FL_PP_), and (FL_PP_ *–* HL_AP_) along the horizontal *x*-axis. The swing phase diagram represents measurements taken from an SCR event.

To investigate limb movement during locomotion, we measured stepping movements of one hindlimb during treadmill locomotion at a speed of 0.3 m/s. The rhythmic movement of the limb was divided into swing and the stance phases. During stance, when the leg provides body support and propulsion, the paw is on the treadmill moving backward relative to the body. During swing, the limb is in the air and moves forward before being placed on the surface to initiate the next stance phase. Throughout the alternating stance and swing phases, the paw moves rhythmically from an anterior to a posterior position, back and forth. The precision of foot placement during locomotion was assessed by kinematic analysis, with the hindlimb anterior position (HL_AP_) defined as the initial point of contact of the toe marker with the treadmill when starting a stance phase, and the forelimb posterior position (FL_PP_) defined as the last contact of the forelimb with the treadmill before initiating the swing phase. The measurements of HL_AP_ and FL_PP_ positions were made using the hip joint as a reference in the *x*-axis (landmark, 0 cm). Forelimb paw position was digitized in its last frame of contact with the treadmill, just before starting a swing phase, and the distance between forelimb paw and the toe marker in the hindlimb was measured (FL_PP_ – HL_AP_; Fig. 1).

**Figure 2.**
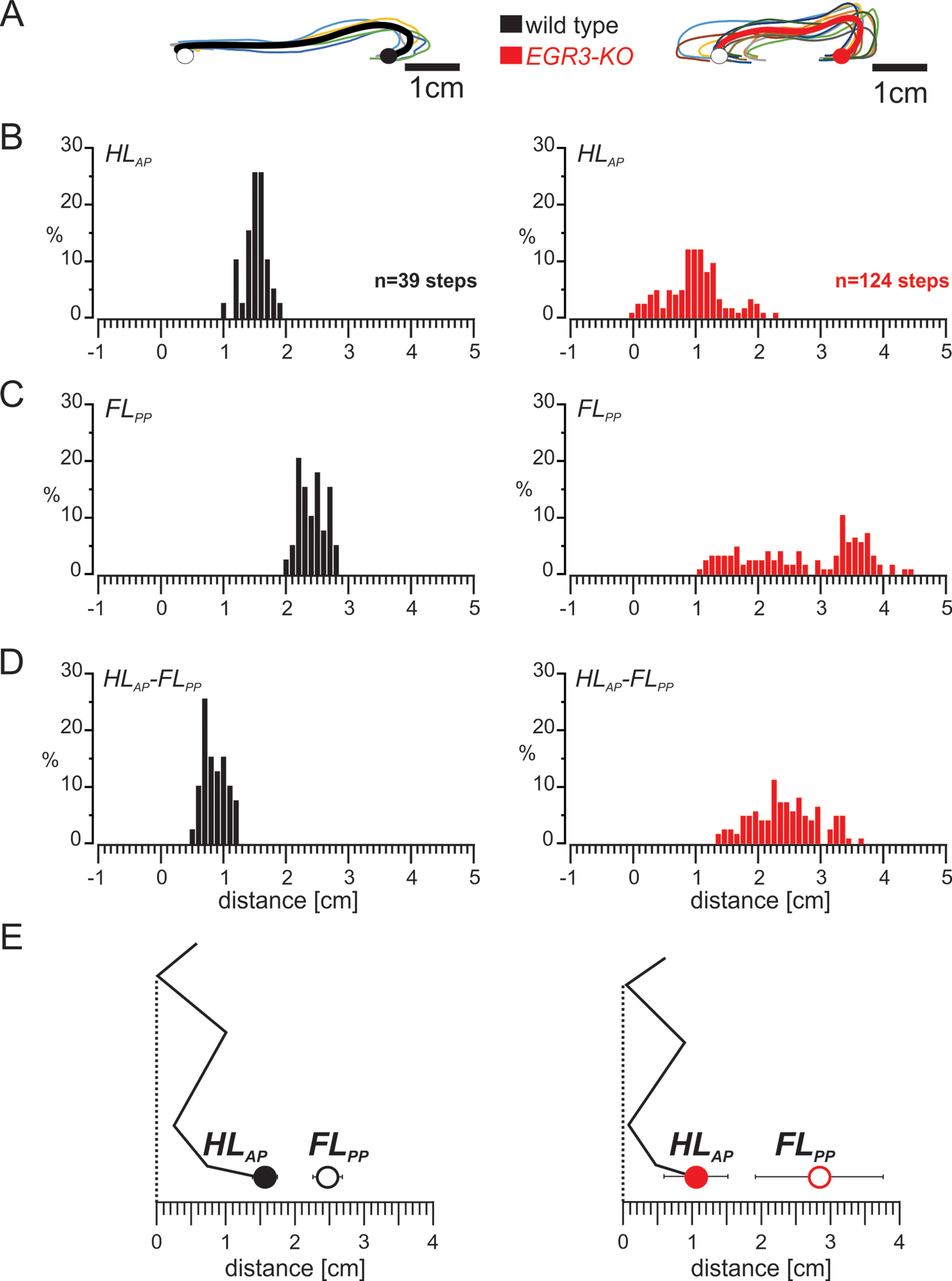
The well organized swing movement in mice requires proprioceptive sensory feedback from the muscle spindles. ***A***, Trajectories of the hindlimb paw of wild-type mice (left) are very stereotyped across animals, whereas more variability in movement is observed in *Egr3-KO* mice (right). The colored lines are swings from an individual animal, and the bold black line is the pooled average. The average posterior paw position at swing onset and the anterior paw position at swing offset are illustrated as open and closed circles, respectively. ***B***, Histogram representing the distribution of hindlimb paw positions relative to the hip joint at the end of hindlimb swing movement (HL_AP_) in wild-type (left) and *Egr3-KO* (right) mice. ***C***, Histogram showing the distribution of forelimb paw positions relative to hip joint at the end of forelimb stance movement (FL_PP_) in wild-type (left) and *Egr3-KO* (right) mice. ***D***, Histogram presenting the distribution of the distance between HL_AP_ and FL_PP_ in wild-type (left) and *Egr3-KO* (right) mice. ***E***, Diagram illustrating the relationship between the average (±SD) of the HL_AP_ and FL_PP_ in wild-type (left) and *Egr3-KO* (right) mice during unperturbed hindlimb swing movements.

We focused our analysis on the swing movement where the hindlimb paw lifts off the ground at the posterior position and is placed back on the anterior position. We observed that in all wild-type animals, this movement occurred consistently through a well defined trajectory (Fig. 2*A*, left) but was less organized and less stereotypic in the *Egr3-KO* mice, which lack muscle spindles (Fig. 2*A*, right). These results suggest that smooth movement of the paw during the swing phase requires proprioceptive sensory feedback from the muscle spindles.

We next investigated the placement of the hindlimb paw relative to the hip joint at the end of the swing movement and its relation to the ipsilateral forelimb paw position at the end of the forelimb stance. We observed that, in general, the distribution of hindlimb paw position (Fig. 2*B*, left), forelimb paw position (Fig. 2*C*, left), and the distance between these positions (Fig. 2*D*, left) were very consistent in wild-type mice. In contrast, these parameters had much higher variability in *Egr3-KO* mice (Fig. 2*B–D*, right). These observations were further confirmed by quantitative comparisons. In wild-type mice, the hindlimb paw was placed on average 1.56 ± 0.18 cm in front of the hip and ∼0.90 ± 0.20 cm behind the forelimb paw, which was on average 2.47 ± 0.21 cm in front of the hip (Fig. 2*E*, left). In *Egr3-KO* mice, the hindlimb paw was placed closer to the hip on average (1.06 ± 0.46 cm in front of the hip; *t* test, *p* < 0.001) and much further from the front paw (2.52 ± 0.53 cm behind the front paw; *t* test, *p* < 0.001; Fig. 2*E*, right). Forelimb paw position at the end of forelimb stance was on average further away from the hip than in the wild-type mice (2.84 ± 0.92 cm in front of the hip joint; *t* test, *p* < 0.001; Fig. 2*E*, right). These data suggest that precise and stereotyped foot placement during locomotion depends on proprioceptive sensory feedback from the muscle spindles.

### Stumbling corrective reaction in the absence of muscle spindles

**Figure 3.**
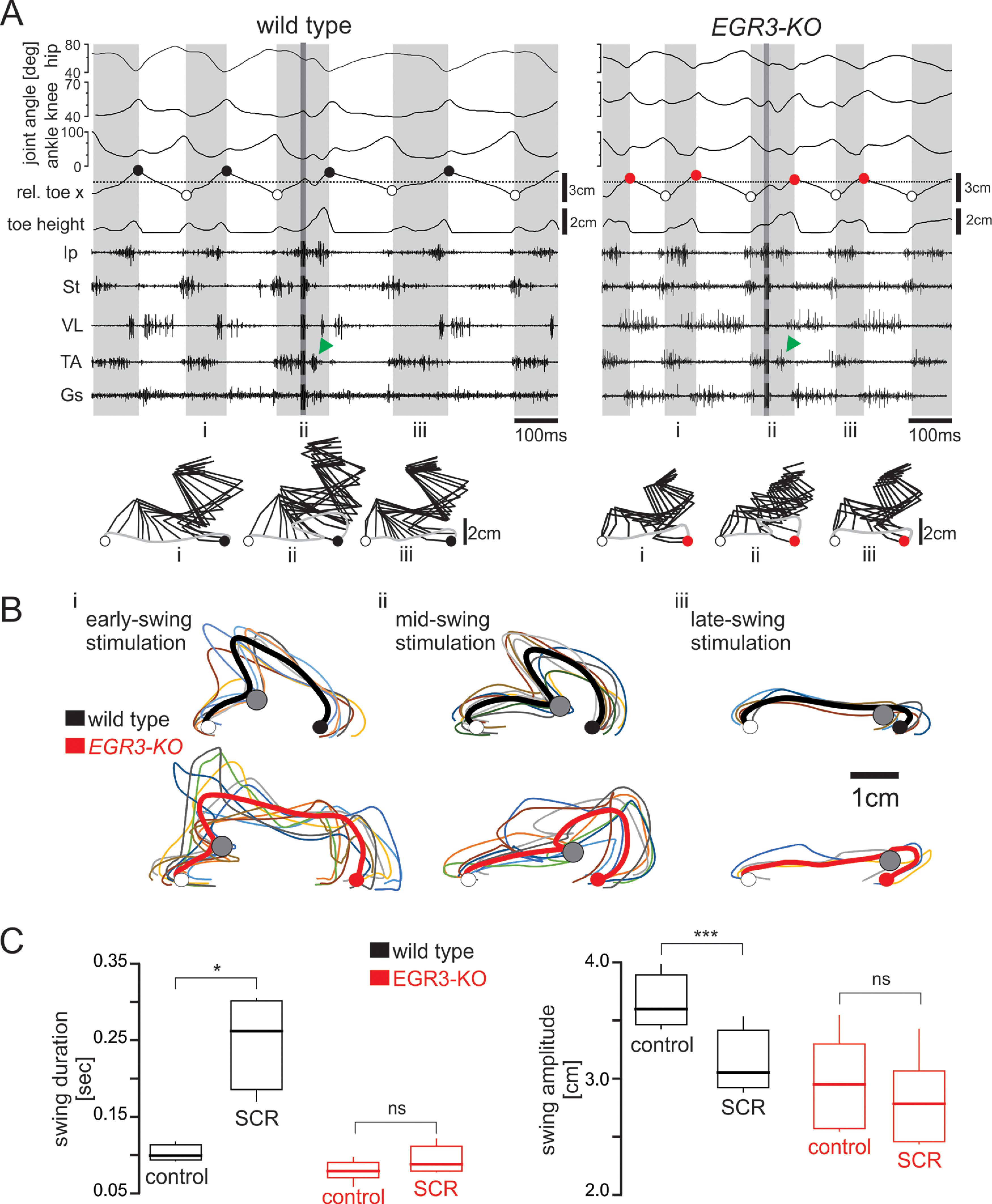
Induction of a stumbling corrective reaction is independent of proprioceptive sensory feedback from the muscle spindles. ***A***, Hip, knee, and ankle joint angles, relative toe *x*- and *y*-coordinates (toe height) synchronized with raw EMG activity of flexor (Ip, St, TA) and extensor (VL, Gs) muscles. Electrical stimulation of the saphenous nerve during swing phase is indicated by the darker gray region inside the third swing phase. Green arrows point to the activity of the flexor muscle elicited by the stimulation. Stick diagram reconstruction of a swing phase before SCR (***i***), an SCR (***ii***), and a swing phase after SCR (***iii***) are illustrated below. ***B***, Trajectories of the hindlimb paw during SCR are very stereotyped in wild-type animals (top) but are disorganized in *Egr3-KO* mice (bottom). The colored lines are individual swings, and the bold line is the pooled average from one animal. The average posterior paw position at swing onset and the anterior paw position at swing offset are illustrated as open and closed circles, respectively. The point of nerve stimulation is indicated by the gray circle. ***C***, Changes in control swing and SCR durations (*p* = 0.016) and distances between the paw position from liftoff to touchdown (swing amplitude) in SCR events in wild-type mice (*p* < 0.001). No changes were observed in swing duration (*p* = 0.074) and swing amplitude (*p* = 0.534) during SCR events in *Egr3-KO* mice.

We next examined how proprioceptive signals control the precision of the swing movements, reasoning that feedback could adjust the movement only at the beginning of the movement, as in humans where vision is used during a critical time window (see Introduction). Alternatively, proprioceptive feedback could regulate hindlimb movement throughout the entire swing movement. To differentiate between these two alternatives, we perturbed the swing movement. We first investigated whether an SCR (Forssberg et al., 1975, 1977; Prochazka et al., 1978; Forssberg, 1979; Mayer and Akay, 2018) could be elicited in the *Egr3-KO* mice that lack muscle spindles. We found that if cutaneous afferent fibers that project through the saphenous nerve were electrically stimulated during an ongoing swing movement in mutant mice, the foot was lifted to clear the virtual obstacle, and this corrective movement was initiated by activation of distal flexor muscles, as in wild-type mice (Fig. 3*A*, Movie 1). However, when we examined the regularity of the paw trajectories during the SCR, we found that the movements looked more irregular in the Eg3-KO mice regardless of whether the stimulation occurred at the beginning or in the middle of the swing phase (Fig. 3*B*, Movie 2). Moreover, when the stimulation occurred toward the end of the swing, no SCR was elicited in *Egr3-KO* mice as it was previously shown with wild-type mice (Mayer and Akay, 2018). Finally, we compared the durations and the amplitudes of the swing movements and the SCR in both groups of mice. In wild-type mice, these spatial and temporal parameters of swing movements were changed during SCR events (duration, *p* = 0.016; amplitude, *p* < 0.001; paired *t* test), whereas in *Egr3-KO* mice, neither swing duration nor amplitude differed (duration, *p* = 0.074; amplitude, *p* = 0.534; paired *t* test; Fig. 3*C*). Our data suggest that proprioceptive sensory feedback from the muscle spindles is not necessary to elicit the SCR, but it is required for timely and well organized execution of the corrective movement.

Movie 1. Stumbling corrective reaction in a wild-type mouse elicited by stimulating the saphenous nerve, indicated by the red circle. 10.1523/ENEURO.0432-21.2021.video.1

Movie 2. Stumbling corrective reaction in an *Egr3-KO* mouse elicited by stimulating the saphenous nerve, indicated by the red circle. 10.1523/ENEURO.0432-21.2021.video.2

### Foot targeting during perturbed swing movement and the role of muscle spindle feedback

**Figure 4.**
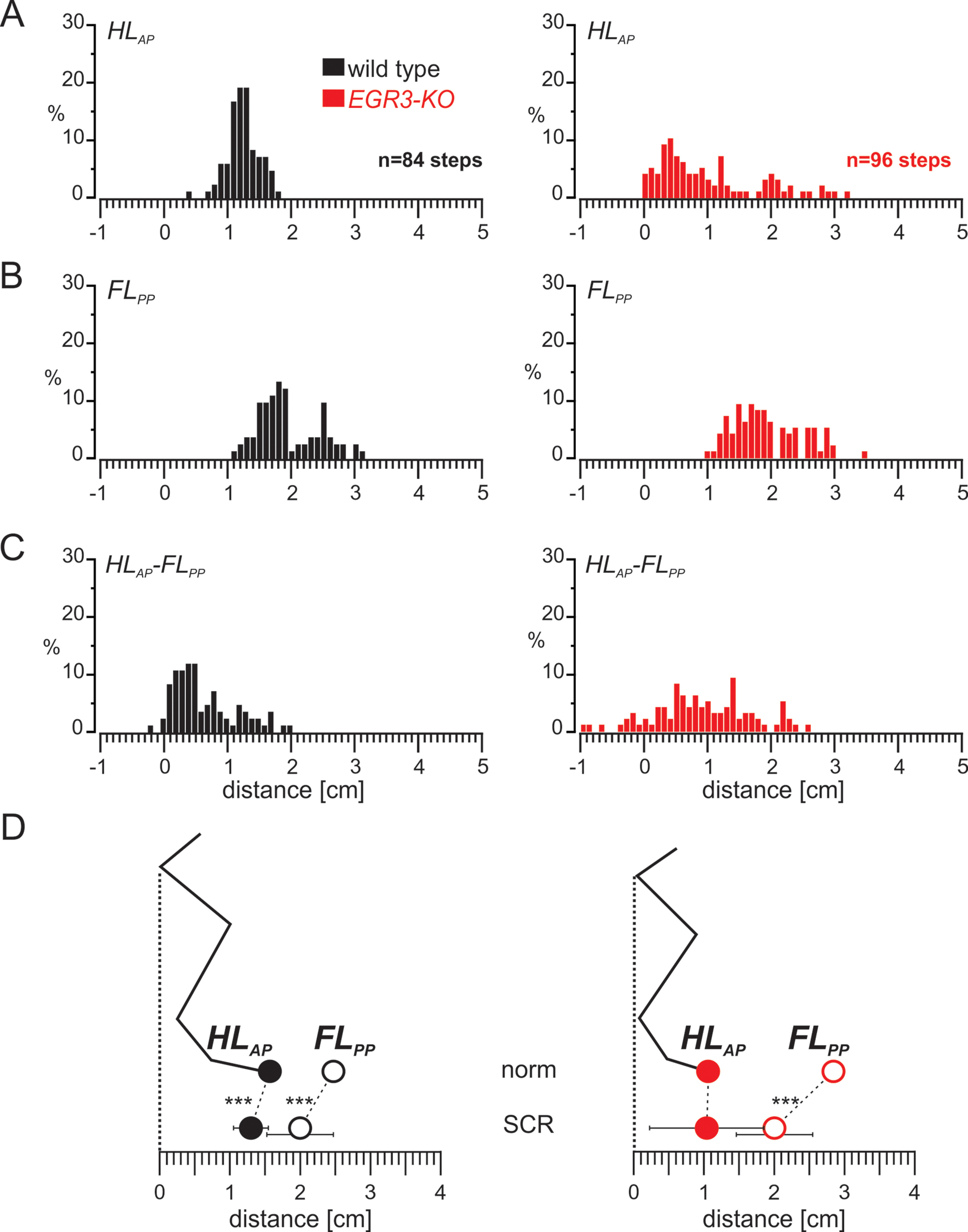
Limb movement during SCR remains well organized in wild-type mice but is more variable in the absence of proprioceptive sensory feedback from the muscle spindles. ***A***, Histogram representing the distribution of HL_AP_ during SCR in wild-type (left) and *Egr3-KO* (right) mice. ***B***, Histogram showing the distribution of FL_PP_ during the SCRs in wild-type (left) and *Egr3-KO* (right) mice. ***C***, Histogram presenting the distribution of the distance between the HL_AP_ and FL_PP_ during SCRs in wild-type (left) and *Egr3-KO* (right) mice. ***D***, Diagram illustrating the relationship between the average (±SD) of the HL_AP_ and FL_PP_ during normal swing movements (norm) and SCRs in wild-type (left) and *Egr3-KO* (right) mice during SCRs. ***p < 0.001. Histograms illustrating the distribution of FL_PP_ during SCR in individual WT and Egr3-KO mice are shown in Extended Data Figure 4-1.

We further investigated the precision of foot placement after SCR in wild-type and *Egr3-KO* mice. In wild-type mice hindlimb paw position was consistently concentrated around a mean value with a narrow distribution (Fig. 4*A*, left). However, forelimb paw position after SCR showed a wider distribution (Fig. 4*B*, left) with two modes, suggesting that there are two types of steps. In the first type, the forelimb stance duration took longer, therefore the paw position moved further back, closer to the hip. In the other type of steps the forelimb stance did not change. To determine whether each mouse displayed this bimodal distribution, we generated histograms from individual animals and confirmed that each wild-type animal contributed to both modes (Extended Data Fig. 4-1). The wider distribution of the forelimb paw position was also reflected in the distance between the hindlimb paw and forelimb paw positions (Fig. 4*C*, left). In the mutant mice, all of these parameters, including hindlimb paw position, were very widely distributed (Fig. 4*A–C*, right) with 10 of 96 steps exhibiting negative hindlimb paw to forelimb paw distance, indicating hindlimb stepping in front of forelimb (Fig. 4*C*, right), which was only observed once in 84 steps in wild types.

10.1523/ENEURO.0432-21.2021.f4-1 Figure 4-1 Histograms of forelimb paw distance from the hip joint of individual wild-type and *Egr3-KO* animals during SCRs. Histograms of FL_PP_ from individual wild-type (left) and *Egr3-KO* (right) animals. In the last histograms on the bottom, all animals are superimposed in a single histogram. Download Figure 4-1, TIF file.

We further confirmed these qualitative observations with quantitative comparisons. In wild-type mice (Fig. 4*D*, left**)**, the hindlimb paw was placed on average 1.3 ± 0.25 cm in front of the hip in a more posterior position than during normal steps (*t* test, *p* < 0.001) with a slightly wider distribution (*F* test: *p* = 0.03). A major difference was observed in forelimb paw position, which was shifted more posteriorly, 1.99 ± 0.47 cm; *t* test, *p* < 0.001), with a higher variability (*F* test: *p* < 0.001) and a bimodal distribution. Moreover, the average hindlimb paw to forelimb paw distance was significantly shorter than during normal steps (0.69 ± 0.5 cm; *t* test, *p* = 0.001) and displayed higher variability (*F* test, *p* < 0.001). Together, these results suggested that during SCR, the relative forelimb paw position at the end of stance was more posterior than in the normal swing. This posterior forelimb paw positioning was accompanied by a posterior shift of the hindlimb paw at the end of hindlimb swing. In contrast, in the absence of muscle spindles, while there was still a posterior shift of the forelimb paw during SCR relative to normal swing, hindlimb position did not shift (Fig. 4*D*, right). This caused the hindlimb and forelimb paws to be placed closer to each other, and in some cases even to be crossed.

### Foot targeting during swing movements during cautious locomotion and the role of muscle spindle feedback

**Figure 5.**
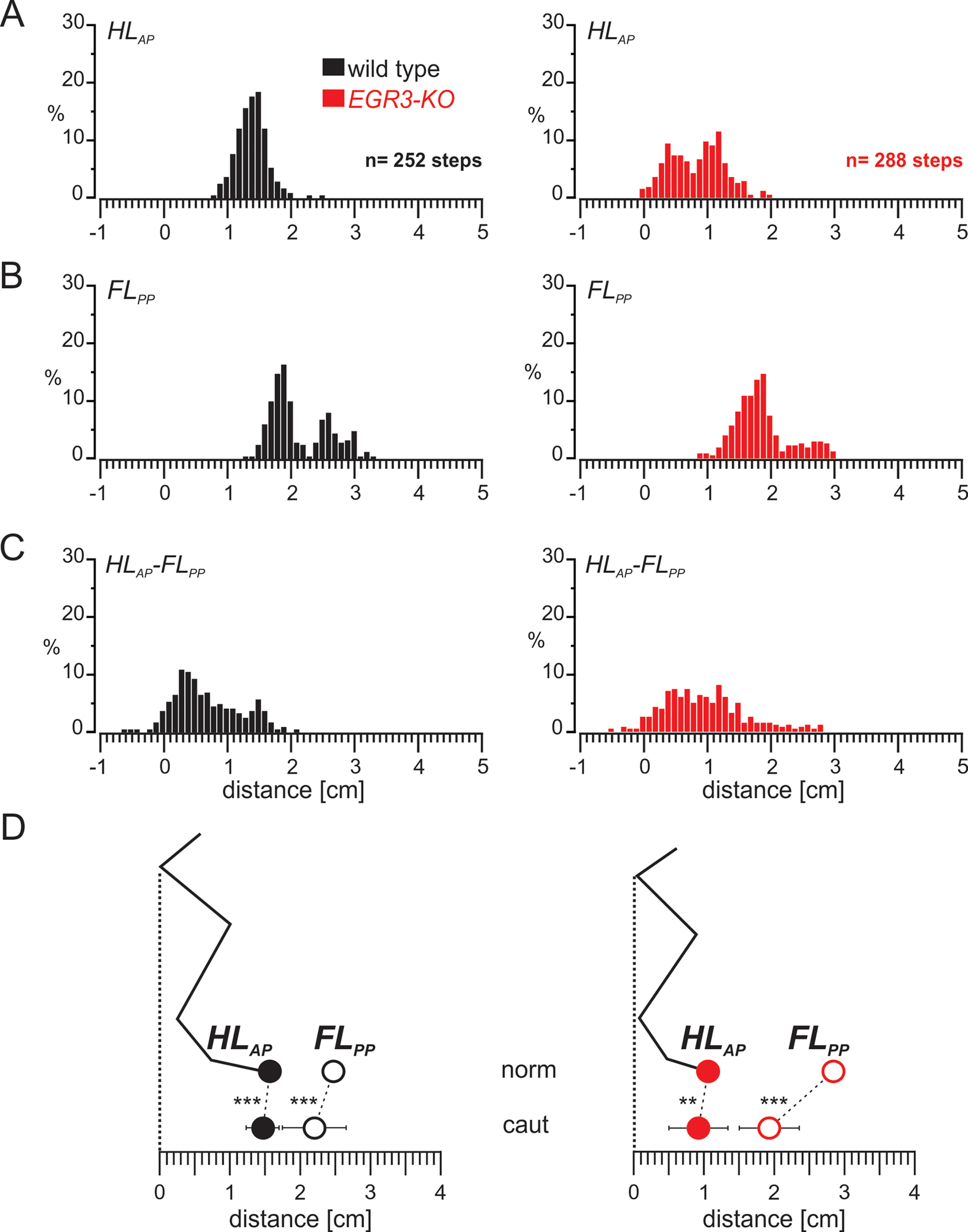
The swing movement during cautious locomotion is well organized in wild-type mice but is more variable in the absence of proprioceptive sensory feedback from the muscle spindles. ***A***, Histogram representing the distribution of HL_AP_ in swing movements during cautious locomotion in wild-type (left) and *Egr3-KO* (right) mice. ***B***, Histogram showing the distribution of FL_PP_ in swing movements during cautious locomotion in wild-type (left) and *Egr3-KO* (right) mice. ***C***, Histogram showing the distribution of the distance between the HL_AP_ and FL_PP_ in swing movements during cautious locomotion in wild-type (left) and *Egr3-KO* (right) mice. ***D***, Diagram illustrating the relationship between the average (±SD) of the HL_AP_ and FL_PP_ during normal swing movements (norm) and swing movements during cautious locomotion (caut) in wild-type (left) and *Egr3-KO* (right) mice during SCRs. **p < 0.01, ***p < 0.001. Histograms illustrating the distribution of FL_PP_ during cautious locomotion in individual WT and Egr3-KO mice are shown in Extended Data Figure 5-1.

We next examined whether these posterior shifts of hindlimb and forelimb paw positions are a direct result of the sensory stimulation causing the SCR, or are they an indirect result of SCRs being elicited? To address these questions, we measured hindlimb and forelimb paw positions during unperturbed steps that occurred between perturbations. Our previous results indicated that these “cautious” steps were different from normal steps but also not the same as SCR (Mayer and Akay, 2018; Santuz et al., 2019). During cautious steps in wild-type mice, the distribution of hindlimb paw position was consistently focused with a narrow distribution (Fig. 5*A*, left), as it was for normal steps and during SCR. However, the forelimb paw position during cautious steps showed a wider distribution clearly exhibiting two modes (Fig. 5*B*, left). Analyzing histograms from individual mice (Extended Data Fig. 5-1), we found that each wild-type animal contributed to both modes. The wider variability in forelimb paw placement was echoed in the distance between the hindlimb paw and forelimb paw positions (Fig. 5*C*, left). In mutant mice, all parameters measured exhibited a wider distribution (Fig. 5*A****–****C*, right). In addition, during cautious locomotion, 8 of 252 in steps in wild-type mice and 5 of 288 steps in mutant mice showed negative hindlimb paw to forelimb paw distance, indicating that the hindlimb was stepping in front of forelimb (Fig. 5*C*, right), as observed during SCR (Fig. 4*C*, right). These results demonstrate that paw position distributions during cautious stepping resemble those observed during SCR.

10.1523/ENEURO.0432-21.2021.f5-1 Figure 5-1 Histograms of forelimb paw distance from the hip joint of individual wild-type and *Egr3-KO* animals during swing movements of cautious locomotion. Histograms of FL_PP_ from individual wild-type (left) and *Egr3-KO* (right) animals. In the last histogram on the bottom, all animals are superimposed in a single histogram. Download Figure 5-1, TIF file.

We further analyzed paw placement values during cautious locomotion quantitatively. In wild-type mice (Fig. 5*D*, left), the hindlimb paw was placed on average 1.45 ± 0.24 cm in front of the hip in a more posterior position than during normal steps but higher than during SCR (*t* test: *p* < 0.001 for both), with a wider distribution than control steps (*F* test, *p* = 0.04) that was similar to results seen during SCR (*F* test, *p* = 0.59). The forelimb paw position was on average shifted more posteriorly (2.19 ± 0.46 cm) compared with control steps (*t* test, *p* < 0.001), but this shift was smaller than that seen during SCR (*t* test, *p* = 0.001), with higher variability than in control steps (*F* test, *p* < 0.001), similar to SCR (*F* test, *p* = 0.70). These shifts resulted in a shorter average hindlimb paw to forelimb paw distance (0.74 ± 0.51 cm) than during normal steps (*t* test, *p* = 0.001) with higher variability (*F* test, *p* < 0.001), similar to SCR (*t* test, *p* = 0.49; *F* test, *p* = 0.78). These results suggest that the parameters of foot placement during cautious locomotion are similar to those measured during SCR, although no perturbation was applied during cautions steps.

We next investigated foot placement parameters during cautious locomotion in *Egr3-KO* mice. We found that, with the exception of the distribution of hindlimb paw placement (Fig. 5*A*), which was wider than in wild-type cautious stepping, other parameters were similar to those in wild-type mice (Fig. 5*B*,*C*). We then quantitatively analyzed the placement parameters. The hindlimb paw was placed 0.90 ± 0.42 cm in front of the hip during *Egr3-KO* cautious locomotion, indicating a posterior shift compared with control steps compared with wild-type mice (*t* test, *p* = 0.001), with a similar distribution (*F* test, *p* = 0.24). Hindlimb paw placement during cautious locomotion was similar to SCR on average (*t* test, *p* = 0.124) with a narrower distribution (*F* test, *p* < 0.001). Forelimb paw placement at the end of the forelimb stance was on average more posterior and had less variability than in control steps (1.92 ± 0.43 cm) away from hip (*t* test, *p* < 0.001; *F* test, *p* < 0.001). When compared with SCR, forelimb paw position in *Egr3-KO* mice was on average similar (*t* test, *p* = 0.179) but slightly less variable (*F* test, *p* = 0.004). As a result, the average distance between the hindlimb and forelimb paws was significantly larger and more variable than during *Egr3-KO* control steps (1.01 ± 0.62 cm; *t* test, *p* < 0.001; *F* test, *p* = 0.035), but was similar to that seen during SCR (*t* test, *p* = 0.55) with less variability (*F* test, *p* = 0.008; Fig. 5*D*, right). These findings demonstrate that, in the absence of proprioceptive feedback from the muscle spindles, the posterior shift of forelimb paw position at the end of forelimb stance is preserved, but the posterior shift of the hindlimb paw at the end of hindlimb swing is compromised during cautious locomotion.

## Discussion

**Figure 6.**
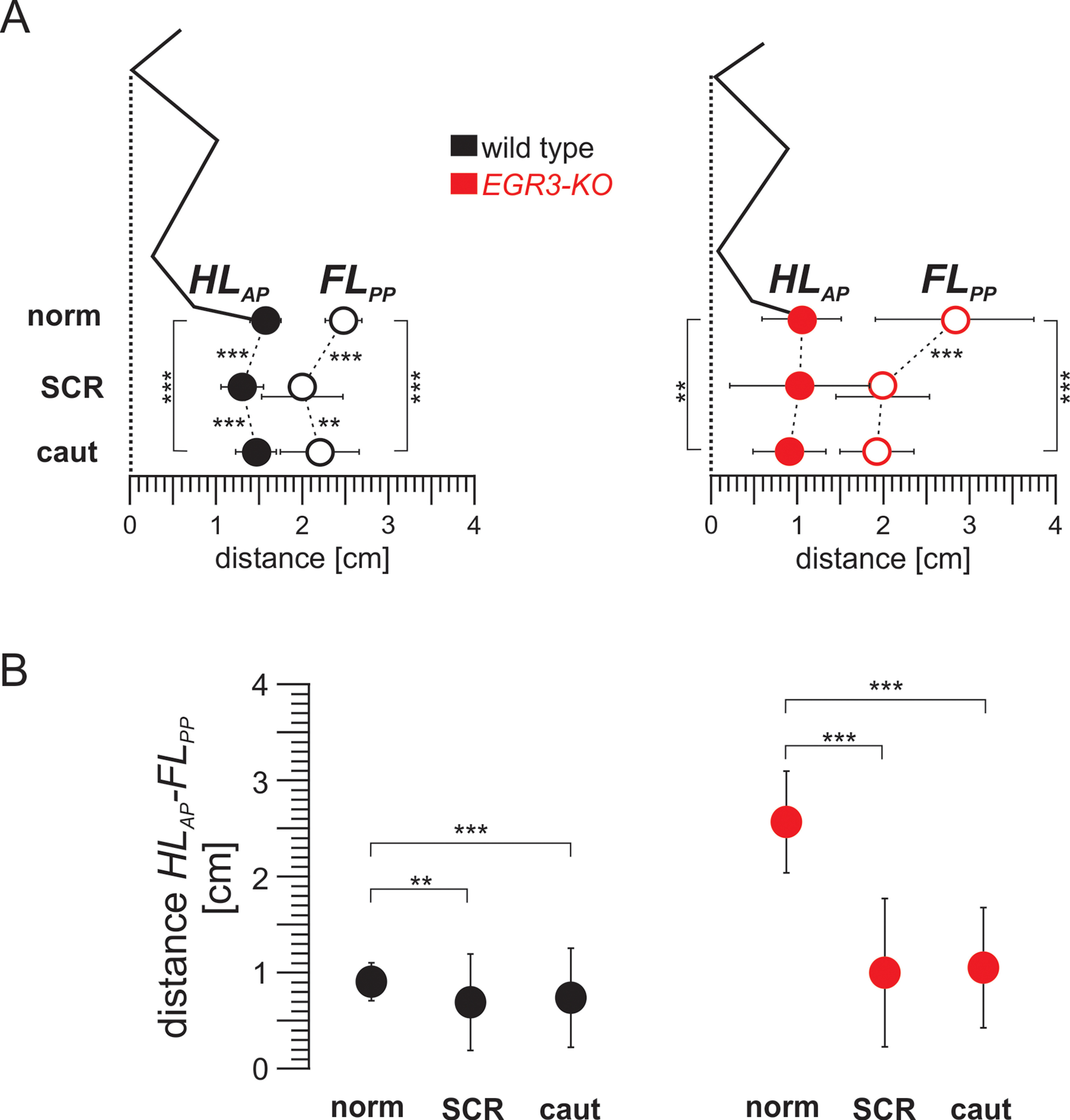
The distance between HL_AP_ and FL_PP_ is relatively constant in wild-type mice but is more variable in the absence of proprioceptive feedback from muscle spindles. ***A***, Diagram illustrating the relationship between the average (±SD) of the HL_AP_ and FL_PP_ in wild-type (left) and *Egr3-KO* (right) mice during control swing movements (norm), SCRs, and swing movements during cautious locomotion (caut). ***B***, Graphs illustrating the average (±SD) of the distance between the HL_AP_ and FL_PP_ in wild-type (left) and *Egr3-KO* (right) mice during control swing movements (norm), SCRs, and swing movements during cautious locomotion (caut). **p < 0.01, ***p < 0.001.

While proprioceptive feedback from the muscle spindles has been shown to be important for foot placement during ladder stepping, whether similar mechanisms exist during normal locomotion on a flat surface was unclear. Our data provide evidence that similar targeting mechanisms are used during locomotion on an even surface, with the hindlimb paw placed just behind the forelimb paw at the end of the swing phase. We further show that when stepping is perturbed by eliciting an SCR, the forelimb paw at the end of forelimb stance shifts posteriorly, followed by hindlimb paw placement also shifting posteriorly (Fig. 6*A*, left). These shifts are not directly related to the SCR itself as they are observed during cautious steps as well when no perturbation is present. Moreover, while the forelimb paw shift during SCR and cautious locomotion is maintained in *Egr3-KO* mice, the posterior shift of hindlimb paw is not present, suggesting that hindlimb, but not forelimb, paw placement is dependent on proprioceptive feedback from muscle spindles (Fig. 6*A*, right). Our data provide insights into the spatial coordination of hindlimb and forelimb placement, and the dependence on proprioceptive sensory feedback from the muscle spindles for precise stepping.

### Swing movement during mammalian locomotion is a targeted movement

Our findings establish that the swing movement during mouse locomotion is a targeted behavior that depends on the proprioceptive sensory feedback from the muscle spindles. Previous research in rats has shown that when an animal walks on a horizontal ladder, the hindlimb is always placed on the same rung that the forelimb was previously placed (Bolton et al., 2006). Later work in mice and rats further showed that this targeted behavior depends on proprioceptive sensory feedback from muscle spindles (Akay et al., 2014; Takeoka et al., 2014; Vincent et al., 2016). However, locomotion on a horizontal ladder is qualitatively a different task than locomotion on a surface (Beloozerova et al., 2010). In contrast to surface walking, walking on a horizontal ladder requires the precise placement of the paw on a rung. In contrast, one could imagine that such precision is not required when the animal walks on a flat surface. It has been suggested based on research with cats and humans that the precision of foot placement at the end of swing might be important for balance control (Klishko et al., 2014; Shafizadeh et al., 2019). Indeed, our data demonstrate an active targeting mechanism while the animal is moving on a treadmill with constant speed, and this mechanism is compromised by the absence of feedback from muscle spindles.

The necessity of proprioception for targeted swing movements was previously shown in insects (Cruse, 1979; Dean and Wendler, 1983; Wosnitza et al., 2013). Here we show a similar mechanism in mice, an evolutionarily distant relative, with the legless urbilateria as the common ancestor from the “Cambrian Explosion” >500 million years ago (De Robertis, 2008). This similarity suggests that although neuronal networks and the legs they control evolved independently, they converged onto the same mechanisms to ensure stable locomotion. These findings suggest a common strategy for locomotor robustness, which might serve as a useful component for bioinspired robots capable of moving robustly through natural environments (Schmitz et al., 2001; Ritzmann et al., 2004; Buschmann et al., 2015).

### Muscle spindles are not necessary for the stumbling corrective reaction

When the leg encounters an obstacle during the swing movement, it generated a well defined SCR, a change of movement that lifts the foot over the obstacle and finishes the swing (Forssberg et al., 1975, 1977; Prochazka et al., 1978; Forssberg, 1979). Is cutaneous afferent stimulation sufficient and necessary to elicit the SCR, as previously suggested (Prochazka et al., 1978; Forssberg, 1979; Mayer and Akay, 2018), or does proprioceptive feedback that signals changes in the natural angular joint movement because of obstacle contact (McVea and Pearson, 2007) also contribute to the initiation of the SCR? Our previous data suggested that cutaneous touch signaling is sufficient to elicit SCR (Mayer and Akay, 2018). Furthermore, the data presented here suggest that muscle spindles are not necessary for the initiation of the SCR. However, we found that muscle spindle feedback is necessary for the smooth trajectory of the paw and the control of foot placement following the SCR. Together, these findings suggest that cutaneous feedback initiates the SCR, but the precision of the movement requires feedback from the muscle spindles.

### Perturbation of swing movement causes the forelimb paw to shift posteriorly

When the swing movement of the hindlimb is perturbed initiating an SCR, the relative position of ipsilateral forelimb paw at the end of the stance shifts posteriorly. This posterior shift of the forelimb paw was also observed in the mutant mice without muscle spindles, suggesting that it is independent of proprioceptive feedback. The following two possible mechanisms could underlie this posterior shift of the forelimb paw. (1) The stimulation of cutaneous afferents that initiates the SCR could also influence supraspinal circuits through ascending pathways (Diaz and Morales, 2016). This information could then be further processed in the brain and finally conveyed to the forelimb to initiate the posterior shift. (2) Alternatively, perturbation could influence the forelimb through mechanical means by shifting the center of mass (Biewener, 2006) and influencing the amount of load the forelimb will be exposed to. This change in load would then be sensed by group Ib afferents from the Golgi tendon organs that are intact in *Egr3-KO* mice (Tourtellotte et al., 2001), causing a delayed onset of swing and therefore a posterior shift of the foreleg paw at the end of the stance. In support of the latter possibility, group Ib afferent signaling has been previously shown to be important for regulating paw position at the end of the stance (Duysens and Pearson, 1980). Our data show that the perturbation-dependent posterior shifts of the hindlimb at the end of the swing and the forelimb at the end of the stance are controlled by different mechanisms, with only the former relying on feedback from muscle spindles.

It is possible that the posterior shift of the forelimb paw is a direct result of the sensory stimulation that initiates the SCR. In this scenario, the SCR elicits an increased swing duration, which would then cause a delayed onset of forelimb swing to increase stability (Park et al., 2019; Latash et al., 2020). Arguing against this possibility, our results indicate that the posterior shift of the forelimb is not caused by the prolonged hindlimb swing during SCR. Indeed, the posterior shift of the forelimb paw at the end of the forelimb stance was also observed during cautious locomotion, when no perturbation to elicit SCR is present (Santuz et al., 2019). These findings suggest that when the possibility of perturbations is present, a cautious locomotion mode is initiated in a feedforward manner to compensate for any possible instability that might occur. Therefore, the posterior shift of the forelimb paw is observed in the absence of muscle spindle feedback and even in the absence of the SCRs being elicited.

In conclusion, we characterized hindlimb paw placement after swing movement as a targeted behavior determined by the forelimb paw position at the end of the forelimb stance. Furthermore, we demonstrated that this targeted swing movement requires proprioceptive sensory feedback from the muscle spindles. That the posteriorly positioned limb is guided by the anteriorly positioned ipsilateral limb through proprioceptive feedback has been shown in insects (Brunn and Dean, 1994; Theunissen et al., 2014). Our observation of a similar mechanism in mammals points to the universality of this mechanism (De Robertis, 2008). Furthermore, these findings provide new opportunities for investigating neuronal mechanisms of targeted forelimb movements during reaching and interaction with objects (Alstermark et al., 1990; Azim et al., 2014).

## Synthesis

Reviewing Editor: Miriam Goodman, Stanford University Decisions are customarily a result of the Reviewing Editor and the peer reviewers coming together and discussing their recommendations until a consensus is reached. When revisions are invited, a fact-based synthesis statement explaining their decision and outlining what is needed to prepare a revision will be listed below. The following reviewer(s) agreed to reveal their identity: NONE. Note: If this manuscript was transferred from JNeurosci and a decision was made to accept the manuscript without peer review, a brief statement to this effect will instead be what is listed below.

Thank you for transferring this manuscript to eNeuro. As editor, I have reviewed the manuscript, prior reviewers, and the authors' response to the reviews. At the editorial level, there are a few revisions to request - please indicate the sex of the mice studied. Thank you!
