# Erratum: Mayer and Akay, “The Role of Muscle Spindle Feedback in the Guidance of Hindlimb Movement by the Ipsilateral Forelimb during Locomotion in Mice”

**DOI:** 10.1523/ENEURO.0531-21.2021

**Published:** 2022-01-21

In the article, “The Role of Muscle Spindle Feedback in the Guidance of Hindlimb Movement by the Ipsilateral Forelimb during Locomotion in Mice,” by William P. Mayer and Turgay Akay, which published online on November 11, 2021, Movie 1 and Movie 2 were inadvertently switched because of a production error. The online version has been corrected.

